# Development of a Pediatric Vascular Catheterization Complication Score (Ped-VCCScore) for predicting post-cardiac catheterization complications

**DOI:** 10.1371/journal.pone.0325044

**Published:** 2025-06-02

**Authors:** Aninthita Praditukrit, Kanjarut Wongwaitaweewong, Pasuree Sangsupawanich, Supaporn Roymanee, Jirayut Jarutach, Rujira Buntharikpornpun, Suppalak Puttharak

**Affiliations:** 1 Department of Pediatrics, Faculty of Medicine, Prince of Songkla University, Songkhla, Thailand; 2 Division of Pediatric Cardiology, Department of Pediatrics, Faculty of Medicine, Prince of Songkla University, Songkhla, Thailand; 3 Division of Pediatric Allergy and Immunology, Department of Pediatrics, Faculty of Medicine, Prince of Songkla University, Songkhla, Thailand; UCSF: University of California San Francisco, UNITED STATES OF AMERICA

## Abstract

Cardiac catheterization, which is essential for clinical diagnosis and treatment, carries certain risks in pediatric patients, including complications such as loss of pulse, internal bleeding, vessel rupture, and subcutaneous hematoma. We investigated vascular complications in pediatric cardiac patients undergoing catheterization and developed a scoring system to predict these risks. We investigated pediatric patients aged <15 years who underwent cardiac catheterization at a tertiary hospital between January 2017 and December 2019 and developed a statistical model identifying the key factors influencing the risk of complications based on complication frequency, patient demographics, and treatment types. The identified key factors were body weight, procedure type, and maximum sheath size in the arterial-side-to-body-weight ratio. Using the scores determined by the model, participants were categorized into low-, intermediate-, and high-risk groups. The effectiveness of the model was assessed based on accuracy, alignment with real outcomes, and the ability to distinguish between cases with and without complications. Of the 390 patients, 6.2% experienced complications after cardiac catheterization. Transient pulse loss was the predominant complication (72%), followed by subcutaneous hematoma (12%) and bleeding (16%). In the development dataset, the vascular complication rates were 1.8%, 6.8%, and 26.1% in the low-, intermediate-, and high-risk groups, respectively. The likelihood ratios for vascular complications in the low-, intermediate-, and high-risk groups were 0.27 (95% confidence interval [CI]: 0.07, 0.81; *P* = 0.014), 1.12 (95% CI: 0.42, 2.67; *P* = 0.828), and 5.38 (95% CI: 2.23, 12.33; *P* < 0.001), respectively. Our model based on body weight, procedure type, and sheath size-to-body weight ratio accurately predicted vascular complications in pediatric cardiac catheterization. As one of the first studies to identify these risk factors, this study highlights the model’s potential applicability to support risk stratification-based clinical decision-making. Further validation in diverse clinical settings is needed to confirm its generalizability and predictive performance.

## Introduction

Cardiac catheterization is a crucial procedure for both the diagnosis and treatment of various congenital and acquired cardiac conditions and involves the insertion of a catheter through arteries or veins, typically accessed via the groin, neck, or arm. In pediatric patients, the risk of vascular complications following catheterization is particularly significant due to their unique anatomical and physiological characteristics, which increase their susceptibility to injuries during the procedure. Vascular complications in pediatric patients range from minor issues, such as temporary pulse loss, to more severe outcomes, including vascular thrombosis or pseudoaneurysms. A thorough understanding of the incidence, types, and associated risk factors is essential for improving patient outcomes and guiding clinical care [1,2].

Despite advances in catheterization techniques, pediatric patients continue to experience complications, such as subcutaneous hematoma (2%), bleeding (3%), and vascular rupture (0.2%) [2]. Pulse loss, a significant complication, has been reported in up to 23% of infants under 1 year old and those weighing less than 9 kg [3,4]. In small pediatric patients, lower body weight and the use of larger arterial catheters are independent risk factors for arterial injury, possibly owing to a mismatch between the catheter size and arterial diameter [5]. Although arterial access using a 4F cannula is generally considered safe in children who weigh 10 kg or less and has a low incidence of serious arterial complications [6], interventional catheterizations confer a significantly higher risk of complications compared with diagnostic procedures. Vascular complications are the most frequently reported, particularly in younger patients (*P* < 0.01) and during interventional procedures (*P* < 0.01) [7]. Thus, these factors are important predictors of the risk of vascular complications and can inform close monitoring in high-risk groups.

Although several risk-prediction tools for post-catheterization complications exist for adults, there is still a lack of structured, pediatric-specific predictive scoring systems. This knowledge gap underscores the importance of developing a tool tailored to pediatric patients to identify high-risk cases and guide clinical decision-making. At our tertiary care center specializing in pediatric cardiac catheterization, vascular complications are frequently observed and represent a major concern. This study aimed to evaluate vascular complications and identify patient and procedural factors associated with increased risk. The primary objective was to determine the incidence of these complications and to develop a predictive scoring system for vascular complications in pediatric patients undergoing cardiac catheterization.

## Materials and methods

### Design, setting, and participants

We conducted a retrospective cohort study with a focus on pediatric cardiac catheterization procedures in September 2021. Data were accessed on January 12, 2022, and all data were fully anonymized before being accessed by the investigator. All data were retrieved from the hospital information system (HIS) of Songklanagarind Hospital between January 2017 and December 2019. Pediatric patients aged less than 15 years who underwent cardiac catheterization were enrolled. During the study period, 471 cardiac catheterization procedure were performed in 429 patients. However, based on the requirement that both the femoral artery and femoral vein should have been accessed during the procedure, only the first procedure for each patient was included in the analysis. A total of 39 cases that did not meet this criterion were excluded, which resulted in a final study population of 390 patients.

### Ethics statement

The study was approved by the Institutional Medical Ethics Committee of Prince of Songkhla University. (REC: 64-331-1-1) and conducted in accordance with the Declaration of Helsinki and the International Conference on Harmonization of Good Clinical Practice (ICH-GCP) guidelines. The Institutional Medical Ethics Committee waived the need of written informed consent for the medical chart review, as the principal investigator also served as the attending physician, and all participants’ identities were anonymized.

### Procedural details

The initial arterial access sheath size was determined percutaneously using the Seldinger technique. Following arterial access, our standard sheath size was a 5-Fr short sheath; however, a 4-Fr sheath was used in neonates. For patients aged <1 year who required initial venous access, a 5-Fr sheath was used, whereas a 6-Fr sheath was used for all patients aged >1 year.

### Post-procedural surveillance

Following the procedure, a dedicated team including an attending physician, pediatric cardiologist, and specialized nurse conducted continuous patient monitoring over a 24-h period. This comprehensive surveillance involved the evaluation of the arterial and venous access points in the recovery ward. Patients were advised to limit physical activity for the initial 6 h, followed by a subsequent examination at 24 h after the procedure.

In our study, post-procedural assessments were standardized, with extremity pulses initially evaluated using palpation and Doppler flow measurements at 30-min intervals for 2 h, followed by an additional assessment after 2 h as part of the standard protocol. If physical examination findings remained within normal limits, pulse monitoring was subsequently reduced to once per nursing shift. In cases where any extremity exhibited signs of coldness or significantly diminished pulses within 1 h following the procedure, immediate interventions were initiated, including warming the affected area and investigating potential vascular complications. If symptoms persisted for more than 2 h, anticoagulation therapy, with a heparin infusion, was administered to maintain the activated partial thromboplastin time (aPTT) at twice the control level.

### Documentation and categorization

All adverse events and their management were meticulously documented until discharge. Vascular access complications were classified into six distinct groups: ecchymosis, bleeding, hematoma, femoral occlusion, pseudoaneurysm, and arteriovenous fistula. The severity of each complication was evaluated using a scale ranging from 1 to 5, following the guidelines set forth by the Congenital Cardiac Catheterization Outcomes Project [8], to which our study strictly adhered. Furthermore, our analysis was extended to procedure-type risk categorization by incorporating insights from the IMPACT Registry Risk Model [9]. Based on these assessments, we categorized the patients into low-, moderate-, and high-risk groups for vascular complications, to facilitate the development of customized, effective prevention strategies for each risk level.

### Definition of vascular complications

In this study, vascular complications were defined as follows. First, ecchymosis (bruising) was defined as the presence of any skin discoloration associated with pain and minor swelling. Second, bleeding was defined as a reduction >2 g/dL from the baseline hemoglobin levels due to blood loss at the puncture site. Third, subcutaneous hematoma was defined as a hard, palpable, and tender swollen mass surrounding the puncture site. Fourth, femoral occlusion (thrombosis) was confirmed by the absence of distal foot pulses and Doppler ultrasound-detected flow, and this was further categorized into transient and complete occlusion of the femoral vessel. Transient occlusion was characterized by the restoration of pedal pulses within a 24-h period, indicating a potential for recovery. In contrast, complete occlusion exhibited no response to treatment. Fifth, pseudoaneurysm was defined as pulsatile mass diagnosed using Doppler ultrasound-detected flow. Lastly, arteriovenous fistula was defined as an abnormal communication between the femoral artery and vein, producing a palpable thrill and bruit sound, confirmed by Doppler ultrasound-detected flow [10].

### Data collection

In this study, the clinical parameters were systematically categorized into three primary groups: basic demographic data, intraoperative data, and adverse event records. The demographic variables included age, sex, and anthropometric measurements. In addition, hemoglobin levels, initial oxygen saturation, and history of catheterization were recorded. Intraoperative data included the treatment modality, fluoroscopy duration, heparin dosage, sheath dimensions, procedure duration, risk categorization, and contrast medium volume. Furthermore, vascular complications occurring intra- and postoperatively were meticulously and extensively documented, whereas vascular access was rigorously monitored within the first 24 h postoperatively. These factors were included in the univariable analysis, and factors that were statistically significant were employed to develop a predictive model for vascular complications.

### Sample size estimation

We estimated the required sample size for developing the multivariable clinical predictive score using the method proposed by Riley et al. [11], which is specifically designed for predictive modeling and serves as a robust alternative to traditional power analysis. This method considers key factors such as the number of candidate predictors, expected outcome incidence, and anticipated model performance. Based on pilot data, we estimated 5 candidate predictors, a C-statistic of 0.85, and an outcome incidence of 5%, which yielded a minimum sample size of 352 with at least 25 events. This approach ensures adequate power for model development, sufficient events per predictor, and limits overfitting while maintaining acceptable precision (≤0.05 margin of error) in estimates of the intercept and adjusted R-squared.

### Statistical analysis

Categorical data are described using frequencies and percentages, and continuous data are described using medians and interquartile ranges (IQR). Descriptive statistics are reported according to the data distribution. We performed an exact probability test to compare categorical data between the two independent groups. In contrast, to compare continuous data, we performed the independent *t* test or Mann–Whitney *U* test, as appropriate.

All statistical analyses were performed using Stata statistical software version 18.0 (StataCorp, College Station, TX, USA), and statistical significance was defined as a two-tailed *P*-value of <0.05.

### Development of the multivariable predictive scores

The parameters with AUC < 0.6 were excluded. Prior to statistical modeling, collinearity among all predictor variables was assessed using variance inflation factors (VIF). Subsequently, a multivariate stepwise forward logistic regression model was constructed to develop a parsimonious model which AUC was comparable to the full model.

To create a risk score, the logit coefficients of each predictor variable were divided by the smallest coefficients and subsequently rounded to the closest integer. Goodness-of-fit (GOF) was quantified using the Hosmer–Lemeshow test and we used bootstrapping to randomize the dataset to internally validate the prediction model. Furthermore, we formulated a calibration plot comparing the agreement between the disease probabilities estimated using the model and the observed disease data.

## Results

**Fig 1:**
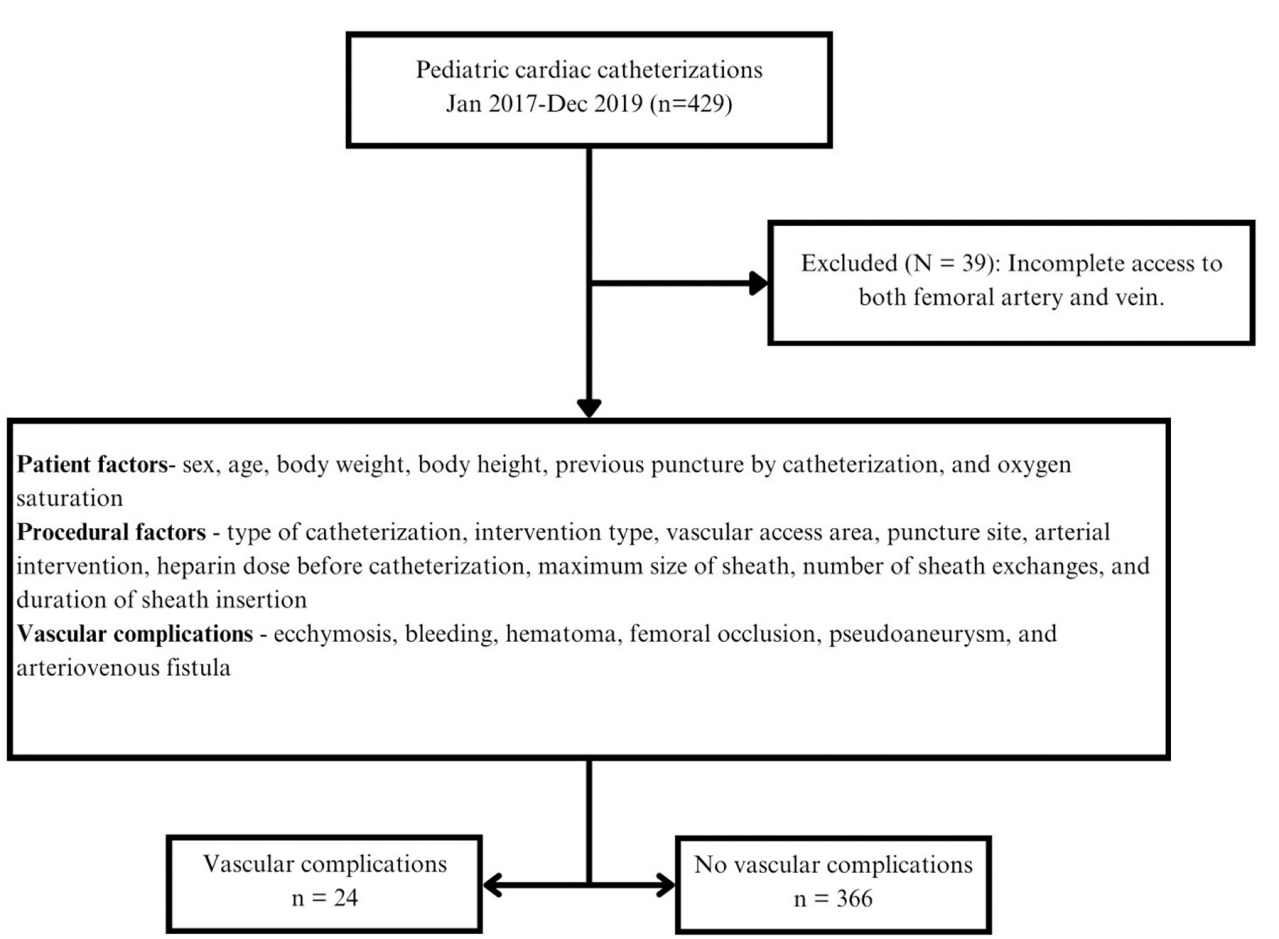
Flow diagram of the patient selection and outcomes.

This comprehensive analysis included 390 participants (Fig 1), of which 207 (53.1%) presented with noncyanotic CHD and 183 (46.9%) presented with cyanotic CHD. In the subset that underwent therapeutic interventions, 148 (64.9%) and 80 (35.1%) patients were diagnosed with non-cyanotic and cyanotic CHD, respectively. Although most participants (309 patients, 79.2%) underwent cardiac catheterization for the first time, 81 patients (20.8%) had undergone the procedure prior to 2017; nonetheless, there was no statistically significant difference between the vascular and non-vascular complication groups with regards to whether catheterization was performed for the first time (*P* = 0.863).

**Fig 2:**
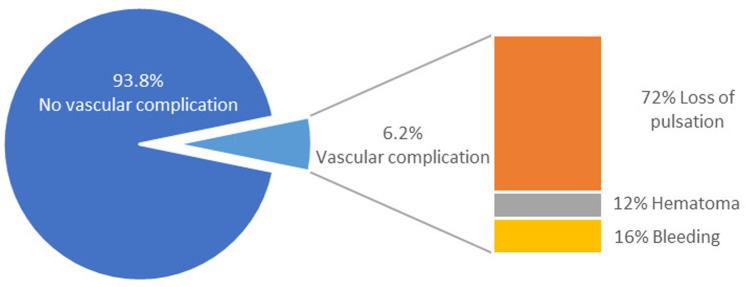
Proportions of the Different Types of Vascular Complications.

In total, 24 (6.2%) patients experienced complications (Fig 2). The predominant complication observed was transient pulse loss, which affected 72% of cases with complications. Other complications included subcutaneous hematoma (12%) and bleeding (16%). Notably, all participants exhibited transient cessation of pulsation postoperatively, negating the need for surgical intervention. In four patients (22.2%), pulsations spontaneously resumed within 4 h postoperatively, while an additional six patients (33.3%) experienced restoration of pedal pulses within 24 h of heparin infusion. However, seven participants required heparin infusions exceeding 24 h, with an average duration of 43 h, resulting in pulse recovery verified through clinical and Doppler examinations. Regrettably, one participant succumbed to hypoxemia and severe acidosis owing to a significant underlying condition.

Based on the severity levels assessed using the 1–5 classification of the Congenital Cardiac Catheterization Outcomes Project [8], the majority of the cohort—comprising 371 participants (95%)—was classified as Level 1, which indicated no harm or alteration in the post-intervention condition. However, nine participants (2.3%) exhibited temporary, non-lethal alterations in their

conditions (Level 2) that were subsequently reversed to their baseline state; eight participants (2.1%) were categorized as Level 3 and experienced serious—albeit transient—changes that necessitated immediate intervention; and one participant (0.3%) had a life-threatening condition (Level 4) that required immediate intervention. Three participants (0.8%) were classified as Level 5, which indicated fatal outcomes during the same hospitalization as that for the cardiac catheterization, although none of these were related to vascular injuries. The first case involved a patient with pulmonary atresia with ventricular septal defect (PA/VSD) who developed thrombosis in a modified Blalock–Taussig shunt (MBTS), which remained occluded despite angioplasty and revision surgery, and resulted in severe cyanosis and death. The second case was of a neonate with obstructive total anomalous pulmonary venous return who, despite a successful balloon atrial septostomy, developed refractory low cardiac output syndrome and died owing to disease progression. The third case was of a patient with right isomerism and nonobstructive total anomalous pulmonary venous return who developed low cardiac output syndrome following diagnostic catheterization and did not survive despite aggressive interventions.

In this study, all 390 patients underwent arterial and venous access procedures. Table 1 provides further details on the analogous patterns observed for variables such as weight, body surface area, and height. Notably, the median age in the complication group was significantly lower than that in the non-complication group (0.5 years vs. 3.8 years, *P* < 0.01); however, univariable analysis revealed no significant sex differences between patients with and without vascular complication. Regarding the procedure type, the incidence of vascular complications was higher in interventional procedures than in diagnostic procedures (*P* = 0.03). Notably, the procedure-type risk categorization demonstrated a higher incidence of complications (*P* < 0.01) in categories 5 and 6 than in the lower categories.

**Table 1:** Baseline characteristics of patients undergoing cardiac catheterization.

Characteristics	Vascular complications (n = 24)	No vascular complications (n = 366)	*P*-value	AUC
Age (years)	0.5 (0.1, 3.3)	3.8 (1.5, 6.7)	<0.01	0.74
Weight (kg)	5.3 (3.0, 10.0)	13.0 (9.0, 18.5)	<0.01	0.77
Height (cm)	59.0 (49.0, 85.5)	96.0 (80.0, 112.0)	<0.01	0.76
BSA (m^2^)	0.2 (0.2, 0.4)	0.5 (0.4, 0.7)	<0.01	0.76
MSA/BW	1.0 (0.5, 1.4)	0.4 (0.3, 0.5)	<0.01	0.78
MSV/BW	1.1 (0.6, 2.0)	0.5 (0.3, 0.7)	<0.01	0.77
Sex (male)	16 (66.7)	182 (49.7)	0.11	0.58
Type of catheterization Diagnosis Intervention	5 (20.8) 19 (79.2)	157 (42.9) 209 (57.1)	0.03	0.61
Procedure-type risk [9] Risk 2 Risk 3 Risk 4 Risk 5 Risk 6	7 (29.2) 2 (8.3) 3 (12.5) 11 (45.8) 1 (4.2)	243 (66.4) 14 (3.8) 34 (9.3) 73 (20.0) 2 (0.5)	<0.01	0.70
Used slender sheath Yes No	9 (37.5) 15 (62.5)	31 (8.5) 335 (91.5)	<0.01	0.64

Data are presented as median (IQR) or n (%); AUC, area under the curve; BSA, body surface area; MSA, maximum size sheath in the arterial side; BW, body weight; MSV, maximum size sheath in the venous side; slender, slender short sheath

Regarding sheath size, the arterial sheath size per body weight ratio was higher in the vascular complication group (1.0 [0.5–1.4]) than in the no-vascular-complication group (0.4 [0.3–0.5]) (*P* < 0.01). Similarly, the venous sheath size per body weight ratio was also higher in the vascular complication group (1.1 [0.6–2.0]) than in the no-vascular-complication group (0.5 [0.3–0.7]) (*P* < 0.01). A larger catheter size is a significant risk factor associated with vascular complications; therefore, a slender sheath is typically required for infants who need a larger-than-usual sheath size. Notably, the use of slender sheaths was four times more frequent in the vascular complication group than in the no-vascular-complication group (*P* < 0.01).

### Model development

Sex was excluded prior to model development owing to an AUC < 0.6. After VIF analysis, three predictive factors (height, body surface area, and MSV/BW) were removed from the model owing to multicollinearity. Consequently, we performed forward stepwise regression to identify a parsimonious model comparable to the full model. The final model included body weight, MSA/BW, and procedure type and achieved an AUC of 0.81—identical to that of the full model. Other potential predictors were excluded owing to a lack of statistical significance (Table 2 and S1 Table).

**Table 2:** Scores assigned using the multivariable logistic regression model for the prediction of vascular complications after cardiac catheterization.

Parameters	Adjusted OR	95% CI	P*-*value	Coefficient	Assigned score
BW (kg) >10 5–10 0–5	1.00 2.30 8.10	– 0.46, 11.54 1.61, 40.89	0.310 0.011	0.835 2.092	0 2 4
Intervention Diagnosis Intervention	1.00 1.74	– 0.57, 5.29	0.326	0.556	0 1
MSA/BW 0.4 >0.4	1.00 2.35	– 0.38, 14.61	0.358	0.856	0 2

OR, odds ratio; CI, confidence interval; MSA, maximum size sheath in the arterial side; BW, body weight

As shown in Table 2, the lowest coefficient was 0.556 (for intervention type), which was assigned a weight score of 1. The coefficient for MSA/BW > 0.4 was 0.856, resulting in a weight score calculated as 0.856/0.556 = 1.54, rounded up to 2. The weight score for body weight was calculated similarly to MSA/BW. Final scores ranged from 0 (indicating lowest risk) to 7 points (highest risk). The scoring criteria were as follows: body weight <5 kg (4 points), 5–10 kg (2 points), or >10 kg (0 points); intervention procedure (1 point) or diagnostic procedure (0 points); and MSA/BW ratio >0.4 (2 points) or ≤0.4 (0 points).

### Score performance

**Fig 3:**
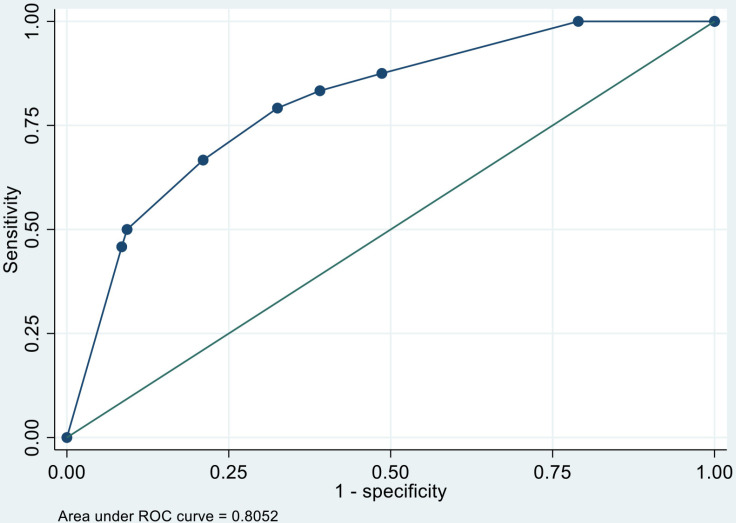
Performance of the Clinical Prediction Score Based on the Area Under the Receiver Operating Characteristics (ROC) Curve. The model incorporates body weight, MSA/BW ratio, and procedure type as independent predictors. The area under the curve (AUC) is 0.8052.

The concordance statistic (C-statistic) calculated using the area under the receiver operating characteristic curve was 0.81 (95% CI: 0.73, 0.90; Fig 3). When performing the Hosmer–Lemeshow test for this model to assess the GOF, we found no evidence of a lack of fit (*P* = 0.862). For internal validation, a bootstrapping method was performed 300 times and showed a consistent area under the curve of 0.80 (95%: CI 0.72, 0.90), with a bootstrap shrinkage value of 1.01.

**Fig 4:**
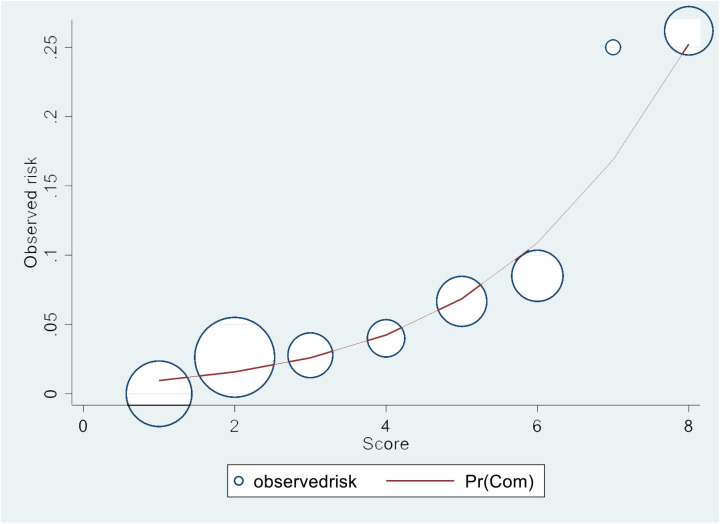
Calibration Curve of the Final Predictive Model for Vascular Complications.

Notably, the risk curve (Fig 4) showed that the predicted risk of vascular complications increased (*y*-axis) in a manner corresponding to an increase in our proposed score (*x*-axis), with the circle size indicating the proportion of patients in each circular area.

The calibration curve demonstrates the agreement between the predicted probabilities of vascular complications (solid red line, Pr (Com)) and observed event rates (blue circles) across risk score categories. The *x*-axis represents the risk scores derived from the final model, whereas the *y*-axis indicates the corresponding observed incidence of complications. The size of each circle is proportional to the number of patients in each risk group, which reflects the distribution of the study population.

### Risk categorization

The vascular complication rates for each score point is shown in S2 Table. Data show similar rate for score 0/2, score 3/5 and score 6/7. Therefore, patients were stratified into three groups based on their assigned scores. Higher scores were associated with a higher prevalence of vascular complications. Table 3 presents the vascular complication rates according to the risk categories based on the predictive scores. In the development dataset, the vascular complication rates were

**Table 3:** Distribution of vascular complications across the low-, intermediate-, and high-risk categories.

Probability categories	Score	Vascular complications (n = 24)		No vascular complications (n = 366)		LHR+	95% CI	*P*-value
		n	%	n	%			
Low	<3	4	1.8	223	98.2	0.27	0.07, 0.81	0.014
Intermediate	3-5	8	6.8	109	93.2	1.12	0.42, 2.67	0.828
High	>5	12	26.1	34	73.9	5.38	2.23, 12.33	<0.001

LHR + , likelihood ratio of positive; CI, confidence interval.

1.8%, 6.8%, and 26.1% in the low-, intermediate-, and high-risk groups, respectively. Moreover, the likelihood ratios for vascular complications in the low-, intermediate-, and high-risk groups were 0.27 (95% CI: 0.07, 0.81; *P* = 0.014), 1.12 (95% CI: 264 0.42, 2.67; *P* = 0.828), and 5.38 (95% CI: 2.23, 12.33; *P* < 0.001), respectively. Using a cutoff of ≥3, the model demonstrated a sensitivity of 83.3%, which indicated a high ability to correctly identify individuals with vascular complications. However, the specificity was 60.9%, which reflected a intermediate ability to correctly identify individuals without complications.

## Discussion

Recent advancements in catheter-based technologies have markedly enhanced the diagnostic and therapeutic modalities for pediatric cardiovascular diseases. The introduction of these methodologies necessitates the rigorous monitoring of their implications and associated risks. Moreover, it is important to note that complications arising from vascular access have consistently represented a significant concern. Our investigation of vascular complications after cardiac catheterization identified adverse events in 6.2% of cases, which aligns with the rates reported in the existing literature, ranging from 3.8%to 32.4% [1,12]. Our results corroborate these findings, demonstrating a significant difference in the median ages of patients with and without vascular complications. Moreover, similar to results found by Roushdy et al., the greatest risk of vascular complications was observed in 32.5% of patients aged <1 year. Subsequent analysis of this study determined that a low body weight and young age serve as principal predictors of severe adverse outcomes, with the highest incidence of complications in patients weighing less than 4 kg (37.5%) [2], followed by those weighing between 4 and 9 kg (29%) and those exceeding 9 kg (17%). Similarly, the mean weight of our patients experiencing complications (5.3 kg) was significantly lower than that of patients without complications (13 kg) (*P* < 0.01). These findings underscore the pivotal roles of age and weight as predictors of vascular complications during pediatric catheterization procedures. Furthermore, they highlight the necessity for heightened vigilance and the development of personalized care strategies to mitigate risks, particularly in susceptible young patients.

Our investigation of vascular complications caused by catheter-based procedures in pediatric populations yielded significant insights. For example, femoral pulse loss was the most prevalent complication, affecting 72% of those with complications, followed by hematoma (12%) and bleeding (16%). These complications are particularly concerning in children aged <10 years [13], with previous research documenting femoral arterial injuries in up to 40% of cases. Glatz et al. reported that the use of larger sheaths, especially catheters of ≥5 Fr, was a significant risk factor associated with a 38% likelihood of acute arterial injury (95% CI: 28%, 48%). Moreover, the ratio of the catheter’s outer diameter to the size of the cannulated artery significantly influences the risk of arterial injury [14]. Our research corroborates these findings, emphasizing the significance of equipment size in patient safety and the efforts required to mitigate these risks. Some studies have identified the number of punctures attempt during cardiac catheterization as a critical determinant of procedural complications, with a significantly higher incidence of complications observed when more than three attempts are required. Specifically, 23% of patients who underwent more than three puncture attempts developed post-procedural arterial complications. However, the retrospective nature of this study introduces inherent limitations, resulting in incomplete documentation of this specific variable [4].

In the present study, we determined that an MSA/BW exceeding 0.4 significantly increased the risk of complications and merited a high-risk score of 2 (Table 2). Conversely, Filis et al.‘s study found no significant correlation between the sheath size and complication rates in adult patients, highlighting the unique vulnerabilities of the pediatric population [15]. In this population, the primary mechanisms of arterial damage include intimal flaps and dissection, which precipitate thrombosis and arterial spasms [16–18]. Moreover, other possible risks include complications such as compression from a large sheath inserted into the femoral vein or arterial spasm caused by accidental puncture of the femoral artery during the establishment of venous access [2,19].

Notably, our data showed that the intervention type was a risk factor associated with vascular complications, with results similar to those of Vitiello et al., [12] confirming that interventional catheterizations exhibit higher vascular complication rates than diagnostic procedures. Previously, Bansal et al. suggested that within a multivariable context, sheath exchanges present the highest risk for arterial damage [20]. In the present study, we found that age, weight, height, the use of a larger sheath size relative to body weight, the type of catheterization, and the procedure-type risk category were independent predictive variables for risk.

Previous studies on pediatric patients undergoing cardiac catheterization, such as IMPACT [9], C3PO [21], and CRISP [22], have significantly advanced our ability to predict adverse events related to congenital cardiac catheterization procedures. Among existing risk models, the IMPACT Registry assesses major adverse events (MAEs) using a robust predictive model (C-statistic 0.76), The C3PO Study categorizes procedural risk but lacks patient-specific predictive capability. The CRISP Score is the most pediatric-focused model, integrating patient and procedural factors to predict severe adverse events (SAEs). However, these models do not specifically address vascular complications, which are the primary focus of our study. For example, Roushdy et al. identified critical risk factors for vascular access complications in pediatric patients, including age < 1 year, body weight <4 kg, prolonged puncture time, and the use of unplanned access sites. Additionally, although the VASCOR score provides a valuable framework for predicting vascular complications in adult patients undergoing interventional cardiology, no comparable model exists for pediatric patients. To address this gap, we introduce the Ped-VCCScore, a dedicated tool designed to assess and stratify vascular risk in children, thereby enhancing patient care, guiding clinical best practices to minimize complications, and optimizing resource allocation by focusing on high-risk groups. Our study specifically focused on vascular complications in this patient population.

**Fig 5:**
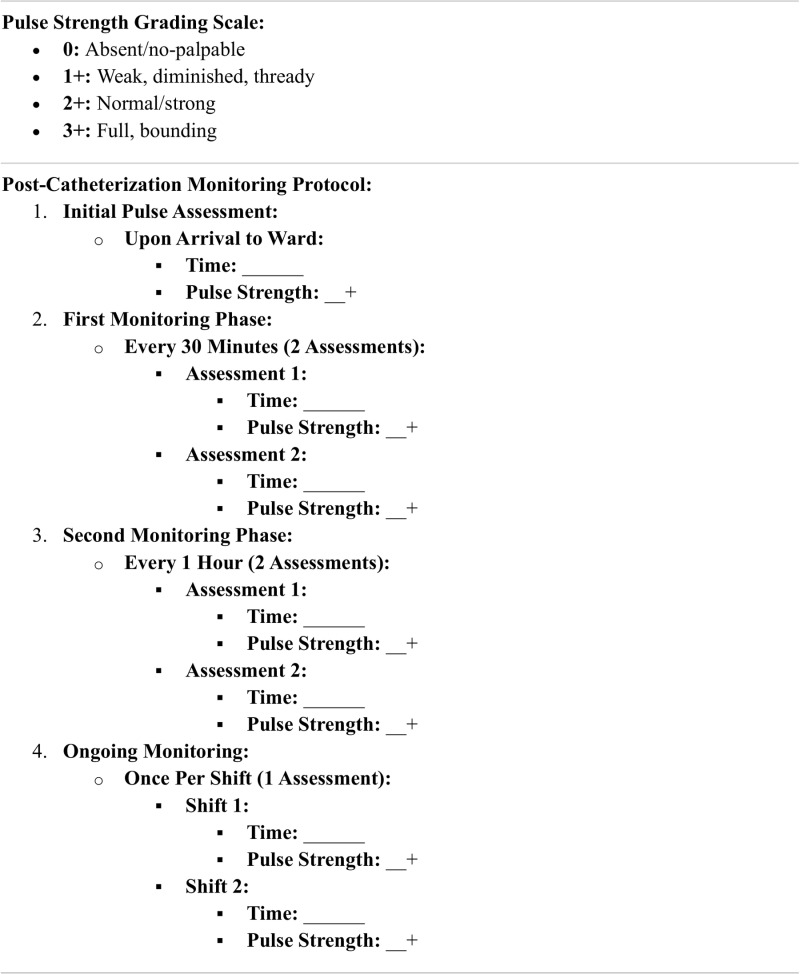
Post-Catheterization Pulse Strength Monitoring Flow.

As outlined in Tables 2 and 3, our final model identified three high-risk predictors for complications. Consequently, this score may provide valuable guidance for healthcare providers in managing post-cardiac catheterization care. The Ped-VCCScore is a 0–7 point system, with patients classified into low (0–2), intermediate (3–5), and high (6–7) risk groups based on similar vascular complication rates. Higher scores correlated with an increased prevalence of vascular complications. Notably, our scoring system demonstrated strong predictive ability for clinical risk, underscoring its practicality as a clinical tool. Patients with a score >5 had a positive likelihood ratio of 5.38 for vascular complications, which indicated a high-risk status that may necessitate close monitoring and special precautionary care. In these cases, the intensive protocol was applied, consisting of evaluations every 30 min for the first two assessments, followed by monitoring every 2 h for the next two evaluations. If physical examination findings remained stable, the frequency of assessments was subsequently reduced to once per nursing shift, as shown in Fig 5.

In this study, we validated the ability of the Ped-VCCScore to resample datasets using bootstrapping statistics, ensuring the validity and reliability of our conclusions. However, the study is limited by its reliance on data from a single institution, which warrants further confirmation regarding the generalizability of the findings to other populations and clinical settings. To address this limitation, we plan to validate the scoring system by using external datasets from another hospital, thereby ensuring its applicability across diverse populations and clinical environments.

Our team proposes a data collection approach that categorizes patients into high-risk, intermediate-risk, and low-risk groups. Patients in the low- and intermediate-risk groups will be managed using the same protocol, whereas those in the high-risk group will receive more intensive care to prevent vascular complications and ensure immediate treatment when necessary.

## Conclusions

The Ped-VCCScore represents a major advancement in the detection of vascular complications during pediatric catheterization, using three key factors for a detailed risk assessment: BW, type of intervention, and MSA/BW. Thus, this study pioneered the identification of novel risk factors for vascular complications and the allocation of weights to these factors according to their aggregate risk. However, further investigation into the development of risk scores such as the Ped-VCCScore is critical for providing targeted care and establishing best practices when performing cardiac catheterization in the pediatric population. Additionally, high-risk patients will benefit from more intensive monitoring to prevent complications and facilitate timely interventions.

## Supporting information

S1 Table Comparison of AUCs between the models. (DOCX)

S2 Table The vascular complication rate for each score. (DOCX)
