# Supplementary material for: Development of a Pediatric Vascular Catheterization Complication Score (Ped-VCCScore) for predicting post-cardiac catheterization complications
Source: PLoS One. 2025 Jun 2;20(6):e0325044. doi: 10.1371/journal.pone.0325044 (PMC12129215; doi:10.1371/journal.pone.0325044)
Supplement: S1 Table — (DOCX) [file pone.0325044.s001.docx]

| **Parameters** | **Odds ratio** | | | |
| --- | --- | --- | --- | --- |
|  | **Model1** | **Model2** | **Model3** | **Model4** |
| BW (kg)  >10  5-10  0-<5 | 1.00  2.78  17.24 | 1.00  4.07  17.43 | 1.00  2.43  10.41 | 1.00  2.30  8.10 |
| MSA/BW  0.4  >0.4 | 1.00  2.33 | - | 1.00  2.12 | 1.00  2.35 |
| Intervention  Diagnosis  Intervention | 1.00  1.74 | - | - | 1.00  1.74 |
| Used slender  short sheath  No  Yes | 1.00  1.49 | - | - | - |
| Procedure-type  risk  2-4  5-6 | 1.00  1.02 | - | - | - |
| Age (years)  >1  0-1 | 1.00  0.37 | - | - | - |
| AUC | 0.81 | 0.78 | 0.79 | 0.81 |

**Supplement 1** Comparison of AUCs between the models
