# Supplementary material for: Development of a Pediatric Vascular Catheterization Complication Score (Ped-VCCScore) for predicting post-cardiac catheterization complications
Source: PLoS One. 2025 Jun 2;20(6):e0325044. doi: 10.1371/journal.pone.0325044 (PMC12129215; doi:10.1371/journal.pone.0325044)
Supplement: S2 Table — (DOCX) [file pone.0325044.s002.docx]

Supplement 2 The vascular complication rate for each score

| Score | Number of patients (N) | Having  vascular complication | |
| --- | --- | --- | --- |
|  |  | N | (%) |
| 0 | 77 | 0 | 0.00 |
| 1 | 114 | 3 | 2.63 |
| 2 | 36 | 1 | 2.78 |
| 3 | 25 | 1 | 4.00 |
| 4 | 45 | 3 | 6.67 |
| 5 | 47 | 4 | 8.51 |
| 6 | 4 | 1 | 25.00 |
| 7 | 42 | 11 | 26.19 |
